# Purification of Indole Contained in Wash Oil by Combination of Extraction and Crystallization (Part 2: Highly Efficient Purification of Indole in Indole-Concentrated Oil via Solute Crystallization)

**DOI:** 10.3390/molecules30112327

**Published:** 2025-05-26

**Authors:** Su Jin Kim

**Affiliations:** Department of Chemical & Biological Engineering, Chungwoon University, Incheon 22100, Republic of Korea; sujkim@chungwoon.ac.kr

**Keywords:** wash oil, indole, extraction, re-extraction, solute crystallization, purification, process

## Abstract

To develop a novel process to purify indole in wash oil with high efficiency, a previous study investigated the indole concentration in wash oil using a process combining methanol extraction and n-hexane re-extraction, which allowed the recovery of a raffinate phase containing 73.3 wt% indole-concentrated oil (ICO). In this study, model ICO, prepared based on the components and compositions present in ICO, and n-hexane were used as the raw material and solvent for the solute crystallization (SC) process, respectively, to examine the effect of SC experimental factors and conditions on the purification of indole. A Pyrex batch crystallizer with a jacket was used as the experimental apparatus. The purity and yield of indole in the generated crystals under optimal SC conditions (crystal washing, a crystallization temperature of 283 K, a crystallization time of 10 min, an impeller speed of 0 s^−1^, and an initial volume ratio of the solvent to model ICO of 15.5) were 99.5 wt% and 57.5%, respectively. Furthermore, this study proposed a novel process for the highly efficient purification of indole contained in wash oil by utilizing the experimental data accumulated from processes in previous research and this study.

## 1. Introduction

Indole, a heterocyclic aromatic compound, has been recognized as a very important intermediate chemical raw material [1] and has wide applications in the synthesis of pharmaceuticals, essential amino acids, and perfumes [2,3,4,5,6,7,8]. Currently, indole is mainly obtained through direct separation from coal tar-based wash oil or crude methylnaphthalene oil [3,4,9], or prepared through chemical synthesis. Although the wt% varies depending on the manufacturer, the wash oil used in this study contained approximately 5.75 wt% of indole. Therefore, the direct separation of indole from wash oil is very valuable in terms of resource recycling and economics.

Methods for separating indole from wash oil have been studied for a long time [10]. At present, acid polymerization and alkaline fusion are widely known as highly effective methods for the direct separation of indole from wash oil [11,12]. However, this method uses both strong acid (e.g., H_2_SO_4_) and strong alkaline (e.g., NaOH) solutions, which is a very inefficient separation method in that it is expensive, severely pollutes the environment, and corrodes metal apparatus. In addition, the separation process becomes very complicated because the separating agent cannot be regenerated, which is a major disadvantage. Supercritical fluid extraction methods [4,9,12], inclusion methods with α-cyclodextrin [13,14], high-pressure crystallization methods [15], ionic liquids methods [16,17,18,19,20,21], and deep eutectic solvent methods (DESMs) [2,5,22,23] have been proposed as alternatives to the acid and base method. However, as disadvantages, these separation and purification methods have relatively low indole purification efficiencies, complex processes, and very harsh conditions, inducing the rapid corrosion of steel equipment. In addition, they use a large amount of diethyl ether, which has very harmful health effects and pollutes the environment. Therefore, it is very important to find an alternative method that can separate and purify indoles contained in wash oil more efficiently and economically.

In the previous study [8], in order to develop a novel process for the highly efficient purification of indole in wash oil to replace the above-mentioned acid–base separation method, a high enrichment of indole was first achieved by combining methanol extraction (ME) and n-hexane re-extraction (HRE) of a batch co-current five-stage (BCC5S) process. This allowed us to recover a raffinate phase (methanol + water + oil) containing approximately 73.3 wt% indole-concentrated oil (ICO).

In this study, the highly efficient purification of indole was examined through solute crystallization (SC) utilizing the differences in melting points and solubility to n-hexane of each component included in the ICO presented in Table 1. The SC experiments investigated the effects of experimental factors (whether or not to wash the generated crystals, crystallization time (t), crystallization temperature (T), impeller speed (N), and initial volume ratio of solvent to raw material (S_0_/F_0_)) and conditions on the purification of indole using a model ICO, which was prepared based on each component and composition present in 73.3 wt% ICO. Additionally, a novel process for the highly efficient purification of indole in wash oil was discussed based on the results of the previous and present studies.

## 2. Experimental Procedure

### 2.1. Manufacturing of Model ICO

The SC raw material, ICO, must be recovered through processes such as distillation from the raffinate phase (methanol + water + ICO), followed by the HRE of BCC5S, as shown in a previous study [8]. However, it was expected that it would take a very long time to recover the amount required for this study. Therefore, this study was carried out by manufacturing a 1 L model ICO based on the composition of each component in the ICO. The model ICO was prepared using four types of heterocyclic aromatic compounds (98 wt% quinoline, 97 wt% iso-quinoline, more than 99 wt% indole, and more than 95 wt% quinaldine) and three types of bicyclic aromatic hydrocarbons (BACs) (95 wt% 2-methylnaphthalene, more than 95 wt% 1-methylnaphthalene, and more than 99 wt% biphenyl) purchased from Sigma-Aldrich Korea. These seven commercial agents used in the manufacturing of the model ICO were used as is without further purification.

Table 1 shows the physical properties and compositions of each component in the model ICO. The composition of indole, the target component for high purification in the model ICO, was 73.3 wt%.

### 2.2. SC Operation

**Figure 1:**
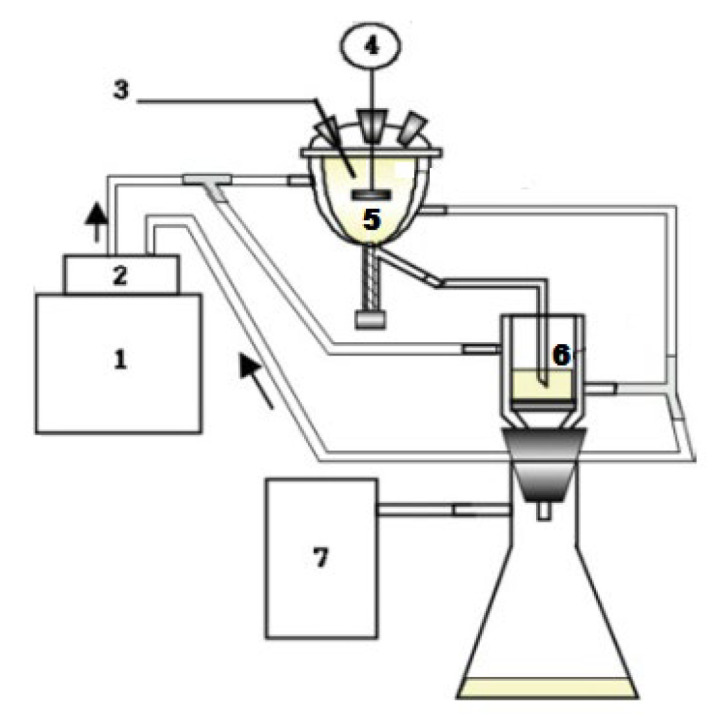
Schematic diagram of batch crystallizer [6]: 1—circulator; 2—PID controller; 3—digital thermometer; 4—variable-speed mixer; 5—crystallizer with a double jacket; 6—glass filter; 7—vacuum pump.

In Figure 1, a schematic diagram of the apparatus used in this study, a batch crystallizer made of Pyrex with an inner diameter of 7 cm and a height of 8 cm is shown [6]. A six-bladed turbine impeller made of stainless steel was used to agitate the solution inside the crystallizer, which was fixed at a position one-third of the way from the bottom of the total solution height. The coolant, cooled to a constant temperature by a PID controller (MC–11, JEIO TECH Co., Ltd., Daejeon, Republic of Korea), was circulated through a jacket installed outside the crystallizer to keep the temperature inside the crystallizer constant. The outside of the jacket was covered with insulation to prevent heat from being transferred from the outside to the crystallizer. The internal temperature of the crystallizer was measured using a digital thermometer (CENTER 300, Center Technology Corp., Taipei, Taiwan).

A certain volume of liquid model ICO melted at 333 K was mixed into a crystallization solvent maintained at 308 K to create a solution with a predetermined volume ratio. This solution was injected into the crystallizer and then cooled (with a cooling rate of 0.5 °C/min) until it reached the specified experimental temperature. When the solution reached the experimental temperature, it was crystallized for a certain amount of time at the set impeller speed, and then the liquid and solid were separated through suction filtration using a vacuum pump (GAST JUN-AIR DOA-P704-AC, IDEX Corp., Michigan City, IN, USA). The generated crystals were either recovered as is without washing or after being washed using a 150 mL washing solvent cooled to the same temperature as the internal temperature of the crystallizer to remove residual impurities attached to the crystals. The recovered wet crystals were placed in a fan-equipped dryer (313 K) to evaporate the n-hexane (crystallization or washing solvent) for 24 h to recover the dry crystals; a small amount of dry crystals was dissolved in acetone and analyzed by gas chromatography (GC), and a small amount of the recovered mother liquor also underwent these processes. The composition of each component contained in the crystals and mother liquor was determined by a calibration curve prepared using the results of the GC analysis of five analytical samples with different compositions; these samples were prepared by dissolving an increasing amount of acetone in a small amount of seven standard reagents, which were selected considering the constituent components of model ICO.

A GC (Hewlett Packard Co., Houston, TX, USA, HP 6890: capillary column, HP-1 (length 60 m, I.D. 0.32 mm, film 0.25 µm)), equipped with a flame ionization detector (FID), as in previous studies [3,8], was used to analyze the samples. The analytical conditions for the samples were as follows: carrier gas, N_2_; volume flow rate, 1 mL/min; sample volume, 1 µL; split ratio, 0.025; injection temperature, 523 K; column temperature, maintained at 383 K for 3 min and then increased at a rate of 5 K/min to 523 K and then 14 K/min to 593 K; and detector temperature, 593 K.

### 2.3. Material System and Experimental Conditions

Table 2 shows the material system and experimental conditions. The model ICO, n-hexane, and 50 vol% methanol aqueous solution, respectively, were used as the raw materials for the SC runs, the crystallization and washing solvent, and the coolant. SC experiments were performed under various conditions for t, N, T, and S_0_/F_0_.

## 3. Results and Discussion

### 3.1. Gas Chromatogram of Model ICO

**Figure 2:**
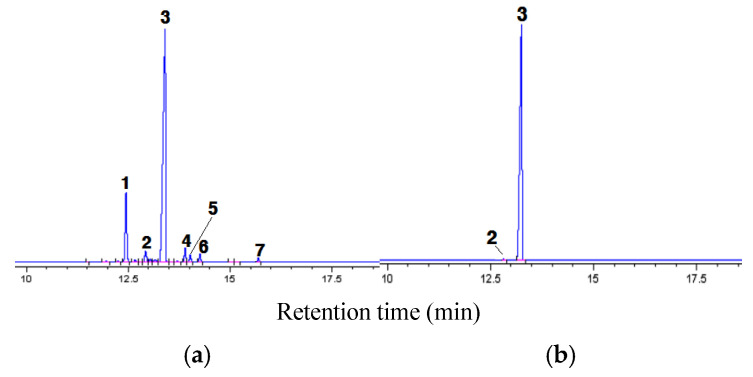
Gas chromatograms of (**a**) model ICO and (**b**) crystal recovered by solute crystallization run using n-hexane. Experimental conditions of (**b**): washing using n-hexane, S_0_/F_0_ = 15.5, T = 283 K, N = 0 s^−1^, and t = 10 min. Peak numbers: 1 = quinolone; 2 = iso-quinoline; 3 = indole; 4 = 2-methylnaphthalene; 5 = quinaldine; 6 = 1-methylnaphthalene; 7 = biphenyl.

Figure 2a shows the gas chromatogram of the model ICO and the component names of the identified components. For the model ICO, indole (peak number 3), the target component for purification in this study, and quinoline (peak number 1) were found in large amounts, while iso-quinoline, quinaldine, 2-methylnaphthalene, 1-methylnaphthalene, and biphenyl were found in very small amounts.

### 3.2. Highly Efficient Purification Performance of Indole

**Figure 3:**
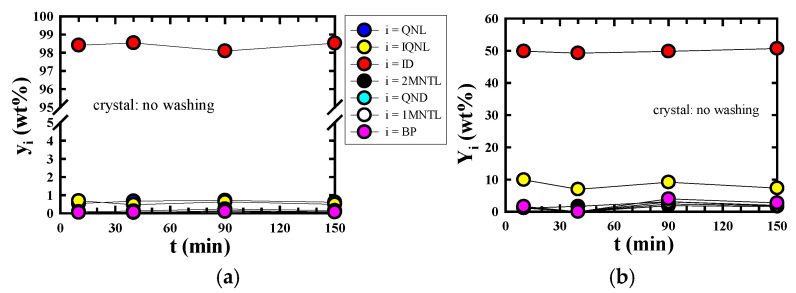
Effect of crystallization time (t) on the (**a**) purity (y_i_) and (**b**) yield (Y_i_) of component i. Experimental conditions: S_0_/F_0_ = 20; T = 283 K; N = 0 s^−1^. Abbreviations: i, component; QNL, quinoline; IQNL, iso-quinoline; ID, indole; 2MNTL, 2-methylnaphthalene; QND, quinaldine; 1MNTL, 1-methylnaphthalene; BP, biphenyl.

Figure 3a,b show the changes with time in the purity (y_i_) and yield (Y_i_) of component i in the crystals recovered, demonstrating the time it takes to reach solid–liquid equilibrium. y_i_ and Y_i_ are the results when the crystals recovered at each crystallization time (t = 10, 40, 90, and 150 min) are not washed with n-hexane. Y_i_ is calculated using the following formula:

$$Y_{i}(\%)=\frac{MC\times y_{i}(g)}{F_{0}\times x_{i,0}(g)}\times 100\%$$

Here, MC and y_i_, respectively, denote the mass of the crystal and the mass fraction of component i in the crystal recovered after the SC run, and F_0_ and $x_{i,0}$ represent the mass and the mass fraction of component i in the model ICO, respectively. y_i_ and Y_i_ showed almost the same value regardless of the operation time. Considering that the solid–liquid equilibrium was reached within 10 min, all SC runs in this study were performed under fixed conditions of t = 10 min. Of the seven components included in the model ICO, indole has a very high melting point (Table 2) and the lowest solubility to n-hexane [8]. Reflecting this, y_indole_ and Y_indole_ were significantly higher than those of the other six components included in the model ICO, regardless of the crystallization time. The resulting crystals had a very low content of impurities such as iso-quinoline.

**Figure 4:**
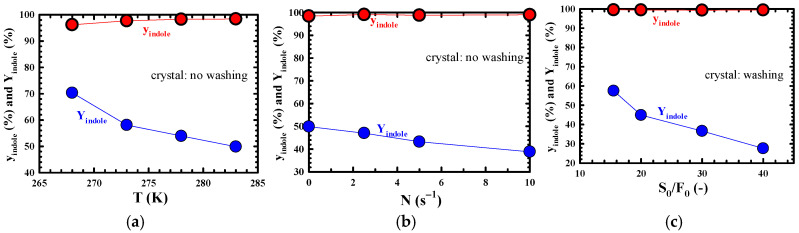
Effect of (**a**) crystallization temperature (T), (**b**) impeller speed (N), and (**c**) initial volume ratio of solvent to model ICO (S_0_/F_0_) on purity (y_indole_) and yield (Y_indole_) of indole. Experimental conditions: (**a**) t = 10 min, S_0_/F_0_ = 20, and N = 0 s^−1^; (**b**) t = 10 min, S_0_/F_0_ = 20, and T = 283 K; and (**c**) t = 10 min, T = 283 K, and N = 0 s^−1^.

Figure 4a–c, respectively, show the effect of T, N, and S_0_/F_0_ on the purity (y_indole_) and yield (Y_indole_) of indole in the crystals. Figure 4a,b show the results when the crystals recovered from each T and N were not washed with n-hexane, whereas Figure 4c shows the results when the crystals recovered from each S_0_/F_0_ ratio were washed with n-hexane. Figure 4a shows the general trend in the SC process where y_indole_ decreases, and, conversely, Y_indole_ increases as T decreases [6]. It is thought that as T decreases, the shape of the crystal breaks and the aggregation of relatively small crystals occurs, causing y_indole_ to decrease as T decreases. On the other hand, as T decreases, solubility decreases, promoting crystallization. This is thought to cause the increase in Y_indole_. In Figure 4b, y_indole_ showed a nearly constant value regardless of N, but B showed a decreasing trend as N increased. The reason why Y_indole_ showed a decreasing trend as N increased is not yet clear, but the reason for this definitely needs to be examined in the future. In Figure 4c, y_indole_ showed almost the same value from 99.3 wt% to 99.5 wt% regardless of S_0_/F_0_, but the increase in S_0_/F_0_ sharply decreased Y_indole_. A decrease in S_0_/F_0_ causes super-saturation. Therefore, it is thought that Y_indole_ increased due to a decrease in S_0_/F_0_. Under the same SC conditions (T = 283 K, t = 10 min, N = 0 s^−1^, and S_0_/F_0_ = 20), as a result of comparing the effect of y_indole_ and Y_indole_ in terms of washing the generated crystals, it was confirmed that y_indole_ was improved after washing (Figure 4c) by about 1 wt% compared to that without washing (Figure 4a,b), but conversely, Y_indole_ was reduced by about 5%. Under the optimal SC conditions (crystal washing, T = 283 K, t = 10 min, N = 0 s^−1^, and S_0_/F_0_ = 15.5), which were chosen considering a balance between y_indole_ and Y_indole_, y_indole_ and Y_indole_ showed very high values of about 99.5 wt% and 57.5%, respectively.

Figure 2b shows the gas chromatogram of a crystal (99.5 wt% indole) recovered under the optimal SC conditions mentioned above. Indole crystals included 0.5 wt% iso-quinoline as an impurity. This allowed us to reconfirm that the n-hexane SC (HSC) process in this study, using n-hexane as a solvent, is a very useful method for the highly efficient purification of indole.

### 3.3. Changes in y_indole_ and Y_indole_ According to Each Process

**Figure 5:**
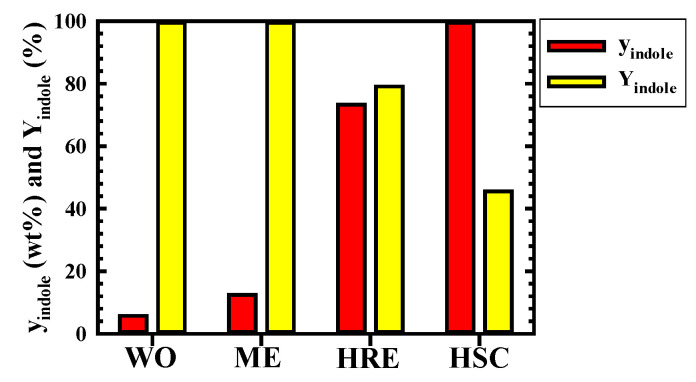
Change in purity (y_indole_) and yield (Y_indole_) of indole obtained by each operation. WO: wash oil [8]; ME: methanol extraction of batch co-current 5-stage process [8]; HRE: n-hexane re-extraction of batch co-current 5-stage process [8]; HSC: n-hexane solute crystallization of batch 1-stage process. Experimental conditions of HSC: t = 10 min, S_0_/F_0_ = 15.5, T = 283 K, and N = 0 s^−1^.

In Figure 5, the changes in y_indole_ and Y_indole_ are investigated and presented according to each process performed in the previous (ME of BCC5S and HRE of BCC5S) [8] and current studies (HSC). Y_indole_ shown in each process is based on the mass of indole in the wash oil. y_indole_ increased rapidly when the following processes proceeded sequentially: (1) crude separation and recovery of indole in wash oil via the ME of BCC5S; (2) enrichment of indole in the methanol extract phase recovered by the HRE of BCC5S; and (3) highly efficient purification of indole in ICO by HSC. The y_indole_ in wash oil was 5.75 wt%, but increased as the process progressed, making the y_indole_ in the crystals recovered after the final HSC process of this study about 99.5 wt%. Y_indole_, on the other hand, decreased rapidly as the process progressed, reaching about 45.5% after the HSC process. It may be possible to improve Y_indole_ by applying multi-step crystallization, recycling the mother liquor, adding an anti-solvent, controlling the degree of supersaturation, and optimizing the crystal particles, etc.

Even when disregarding the effect of the type of raw material on the y_indole_ of the final product (crystal), in this study, the final y_indole_ equal to 99.5 wt%, obtained using wash oil containing 5.75 wt% indole produced directly from a coal tar manufacturing company, was significantly higher than the 98.6 wt% and 98 wt% obtained by high-pressure crystallization [15] and supercritical fluid extraction [4] using model mixtures containing 69.3 wt% and 10 wt% indole. Recently, the DESMs, using a model wash oil containing 25 wt%, indole was reported to achieve y_indole_ = 96.63 wt% and Y_indole_ = 46.02% [5]. The methods in this study were compared with the DESMs, showing that the y_indole_ of this study was 2.87 wt% higher than that obtained by the DESMs. However, since Y_indole_ showed almost the same value in both purification processes, it was expected that the process in this study would be more effective for the highly efficient purification of indole compared to that of the DESMs.

### 3.4. Proposal of a Novel Process for the Highly Efficient Purification of Indole in Wash Oil

**Figure 6:**
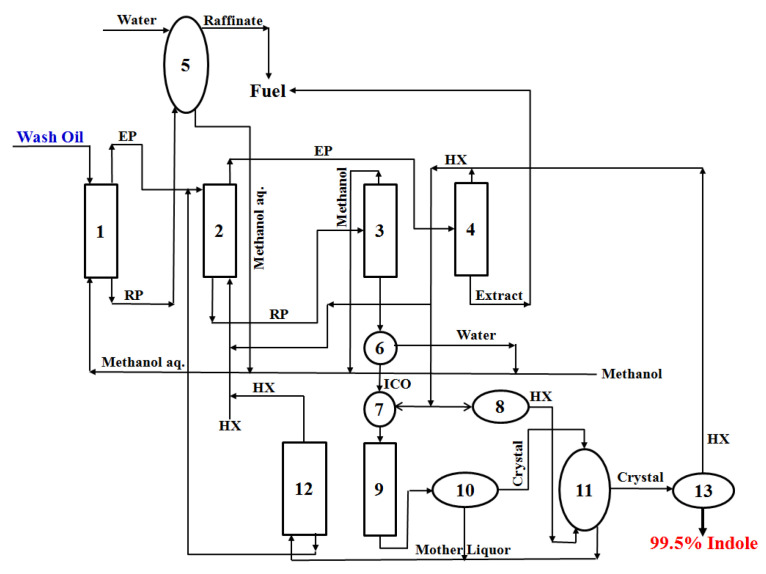
Highly efficient purification process for indole in wash oil. EP: extract phase; RP: raffinate phase; aq.: aqueous solution; HX: n-hexane; ICO: indole-concentrated oil. Device numbers: 1—extraction tower; 2—re-extraction tower; 3, 4, and 12—distillation tower; 5—washing tower; 6—settling tank; 7—solution manufacturing tank; 8—cooling unit; 9—crystallization tower; 10—filtration unit; 11—washing unit; 13—drying unit.

Using only experimental data on the separation and purification performance of indole accumulated from a previous study [8] and this study, the separation and purification process of indole in wash oil was examined. Figure 6 shows the process proposed via this study, which consists of one extraction tower, one re-extraction tower, three distillation towers, one washing tower, one crystallization tower, one settling tank, one solution manufacturing tank, one cooling unit, one filtration unit, one washing unit, and one drying unit. Towers 1 and 2 represent an extraction tower for recovering an extract phase containing indole from wash oil and a re-extraction tower for recovering a raffinate phase containing ICO, respectively. Towers 3, 4, and 12, respectively, are distillation towers for recovering the extraction solvent (methanol), re-extraction solvent (n-hexane), and washing solvent (n-hexane) for washing the generated crystals. Tower 5 is the washing tower for recovering the methanol contained in the raffinate phase. Tanks 6 and 7 represent a settling tank for recovering ICO through phase separation between water and ICO and a solution manufacturing tank using ICO and SC solvent for the SC process, respectively. Tower 9 is a crystallization tower for the highly efficient purification of indole. Units 8, 10, 11, and 13, respectively, are a cooling unit for cooling n-hexane, a filtration unit for solid–liquid separation, a washing unit for washing the generated crystals, and a drying unit (313 K) for drying wet crystals.

Based on the process proposed in this study, a more precise process design will be required in the future by examining the scale-up of process units, fixed and energy costs, and quantitative modeling.

## 4. Conclusions

The highly efficient purification of indole in wash oil was investigated via a combination of ME–HRE–HSC processes. As these processes progressed, the concentration of indole in wash oil was 5.75 wt%, but the purity of indole in the crystals recovered through the final HSC process of this study and the yield based on the mass of indole contained in the wash oil were 99.5 wt% and 45.5%, respectively. Furthermore, a novel process was proposed for the highly efficient purification of indole in wash oil by synthesizing the experimental data accumulated through ME, HRE, and HSC. The proposed process is expected to be a high-efficiency process that can be utilized as an alternative to the current acid–base process.

## Figures and Tables

**Table 1:** Physical properties and compositions of each component in the model ICO.

Compound	Physical Property		Composition (wt%)
	b.p. * [K]	m.p. ** [K]	Model ICO
Quinoline (C_9_H_7_N)	511	257	14.34
Iso-quinoline (C_9_H_7_N)	515	299–301	2.59
Indole (C_8_H_7_N)	526	325	73.31
2-Methylnaphthalene (C_11_H_10_)	514–515	307–309	2.58
Quinaldine (C_10_H_9_N)	521	271	1.36
1-Methylnaphthalene (C_11_H_10_)	513–516	251	1.56
Biphenyl (C_12_H_10_)	528	342	0.80
Others			3.46

*: boiling point, **: melting point.

**Table 2:** Material system and experimental conditions.

Material System	
Raw material	model indole-concentrated oil (model ICO)
Crystallization solvent	n-hexane
Washing solvent	n-hexane
Coolant	50 vol% methanol aqueous solution
Experimental conditions	
Crystallization time, t [min]	10–150
Crystallization temperature, T [K]	268–283
Impeller speed, N [s^−1^]	10–150
Initial volume ratio of solvent to model ICO, S_0_/F_0_ (-)	15.5–40

## Data Availability

The data presented in this study are available within the article (tables and figures).
